# Pathway-Level Reorganization of Genetic Signals Associated with Low Bone Mineral Density Across the Menopausal Transition

**DOI:** 10.3390/ijms27104447

**Published:** 2026-05-15

**Authors:** Soo-Eun Choi, Su Kang Kim, Gyutae Kim, Ju Yeon Ban, Sang Wook Kang

**Affiliations:** 1 Department of Anesthesiology, Cheonan Chungmu Hospital, Cheonan 31144, Republic of Korea; kiroroe07@naver.com; 2 Department of Biomedical Laboratory Science, Catholic Kwandong University, Gangneung 25601, Republic of Korea; skkim7@cku.ac.kr; 3 Department of Oral and Maxillofacial Radiology, College of Dentistry, Kyung Hee University, Seoul 02447, Republic of Korea; latinum.omfr@khu.ac.kr; 4 Department of Dental Pharmacology, College of Dentistry, Dankook University, Cheonan 31116, Republic of Korea; jyban@dankook.ac.kr; 5 Department of Oral and Maxillofacial Pathology, College of Dentistry, Kyung Hee University, Seoul 02447, Republic of Korea

**Keywords:** menopausal transition, low bone mineral density, cross-platform convergence, genome-wide association study (GWAS), pathway analysis

## Abstract

Osteoporosis in women is strongly influenced by menopause, a major physiological transition that reshapes bone metabolism. Although low bone mineral density (BMD) in premenopausal women and osteoporosis in postmenopausal women share the clinical outcome of skeletal fragility, it remains unclear whether they reflect a shared molecular program or distinct regulatory mechanisms. Here, we compared genetic signals associated with premenopausal and postmenopausal low BMD in Korean women using two independent genotyping platforms with distinct variant coverage. After allele harmonization and heterogeneity testing, variants were classified as reversal signals, showing directionally discordant effects across menopausal status, or stable signals, showing concordant effects. Gene-level association analysis was performed using Multi-marker Analysis of GenoMic Annotation (MAGMA), followed by functional enrichment and network-based analyses. Reversal and stable signals showed distinct biological patterns. Reversal signals consistently converged on cyclic nucleotide-related pathways, including cyclic adenosine monophosphate/cyclic guanosine monophosphate (cAMP/cGMP) signaling and nitric oxide-mediated processes, whereas stable signals were more broadly distributed across pathways related to ion homeostasis, cell–substrate adhesion, and structural maintenance. These pathway-level patterns were reproducible across platforms despite limited SNP-level overlap. These findings suggest that low BMD across the menopausal transition is better resolved at the gene and pathway levels than at the level of individual SNPs.

## 1. Introduction

Osteoporosis is one of the most important chronic musculoskeletal disorders in women and poses a major public health challenge. Its consequences go far beyond a simple reduction in bone density, leading to fragility fractures, functional decline, poorer quality of life, and higher mortality. Women carry a disproportionately greater burden of osteoporosis and osteoporotic fractures than men, largely because the rapid bone loss that occurs after menopause is a major risk factor. Recent clinical guidelines and epidemiologic studies likewise regard osteoporotic fractures as major clinical events in women’s health, with hip fractures imposing a particularly high burden of morbidity and disability [1].

In women, menopause is not simply a consequence of aging but a major physiological turning point that fundamentally alters bone homeostasis and remodeling. Estrogen deficiency increases bone resorption and disrupts the balance between bone formation and resorption, leading to accelerated bone loss during the menopausal transition and soon after menopause. Long-term follow-up studies also suggest that rapid trabecular bone loss may already begin during the menopausal transition, before estrogen is fully depleted. From this perspective, separating premenopausal and postmenopausal stages is not just a way of dividing study populations but also an important analytical framework for capturing a key biological shift in bone metabolism [2].

Thus, we separately investigated genetic signals associated with low low bone mineral density (BMD) in premenopausal and postmenopausal women. In previous studies, we identified genome-wide association signals related to osteoporosis risk in Korean premenopausal women [3] and, in Korean postmenopausal women, reported candidate variants and linkage disequilibrium (LD)-defined loci by integrating data from two exome-based genotyping platforms [4]. Collectively, these findings support the involvement of genetic factors in susceptibility to low BMD or osteoporosis across both premenopausal and postmenopausal women.

However, low BMD in premenopausal women and osteoporosis in postmenopausal women are not biologically identical states. In premenopausal women, low BMD has a different relationship with fracture risk and requires a different interpretive framework, with greater emphasis on identifying secondary causes and interpreting bone status on the basis of Z-scores. Even so, the two conditions still converge at the level of skeletal phenotype, as both are associated with low bone mass, reduced bone strength, and greater long-term fragility. From this perspective, bone disease in premenopausal and postmenopausal women is better understood not as exactly the same condition or as two completely unrelated entities, but as part of a continuum in which menopause introduces an additional layer of molecular and physiological reorganization on a shared biological background [5].

From these observations, the central question of the present study emerges. Because menopause represents a major physiological transition in bone metabolism, it is plausible that low BMD before and after menopause is influenced, at least in part, by distinct genetic and molecular pathways [6]. At the same time, since both premenopausal low BMD and postmenopausal osteoporosis converge on the clinical outcome of skeletal fragility, certain biological pathways may be preserved across menopausal status. However, despite this shared endpoint, the underlying metabolic dynamics and structural phenotypes differ substantially, suggesting that similar clinical manifestations may arise through partially distinct biological mechanisms [7]. Existing studies have more often examined premenopausal and postmenopausal women separately or identified risk loci at specific clinical time points. Although these approaches have yielded important insights, they provide only limited leverage for systematically distinguishing which biological axes are preserved, which are reorganized, and which might show directionally distinct effects across the menopausal transition.

Furthermore, assessing commonalities between premenopausal and postmenopausal groups based only on direct single-nucleotide polymorphism (SNP) overlap has important limitations. Different genotyping platforms capture different sets of variants, and the same biological process may be represented by different variants that ultimately map to the same gene or pathway [8]. As a result, focusing solely on SNP-level overlap may underestimate shared biology. A more integrative approach—combining SNP-, gene-, and pathway-level analyses—is therefore needed to better characterize both shared and distinct mechanisms across menopausal stages [9,10].

This study was undertaken to address that gap. First, we comprehensively compared pathway-level genetic signals associated with low BMD in premenopausal and postmenopausal women in order to identify biological pathways that are preserved across menopausal status. Second, by identifying pathways that differ across the menopausal transition, we sought to distinguish a shared biological basis of osteoporosis in women from molecular programs that are more specific to menopause-related changes. Third, we examined whether different variants or genotyping platforms converge on the same functional pathways, with the goal of capturing higher-order pathophysiological structure beyond simple SNP-level discordance. Ultimately, we aimed to provide a basis for viewing osteoporosis in women not as two completely separate conditions before and after menopause, but as a continuous molecular spectrum in which commonality and divergence coexist.

## 2. Results

### 2.1. Analytical Workflow Identifies Direction-Specific Genetic Signals Across Datasets

**Figure 1:**
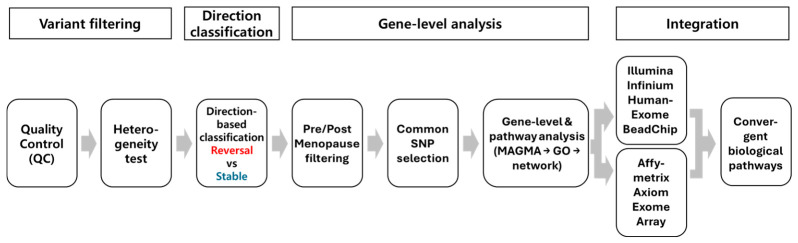
Analytical workflow for reversal and stable genetic signals across Illumina Infinium HumanExome BeadChip and Affymetrix Axiom Exome Array datasets. Schematic overview of the study pipeline. After quality control and heterogeneity testing, variants were classified into reversal and stable groups according to effect direction and heterogeneity. Retained variants from the pre- and postmenopausal analyses were mapped to genes using MAGMA. Functional annotation, pathway enrichment, and gene–concept network analyses were performed separately using the Illumina Infinium HumanExome BeadChip and Affymetrix Axiom Exome Array datasets, followed by cross-platform comparison. Red font indicates reversal signals, whereas blue font indicates stable signals.

To investigate genetic effects associated with menopausal transition, we implemented an integrated multi-step analytical framework combining Illumina Infinium HumanExome BeadChip and Affymetrix Axiom Exome Array datasets (Figure 1). Following stringent quality control and heterogeneity testing, genetic variants were classified according to the direction of effect into reversal and stable groups. In this study, “reversal” and “stable” signals were defined according to the directionality and heterogeneity of SNP-level effects between premenopausal and postmenopausal groups. Reversal SNPs were defined as variants showing opposite directions of effect between the two groups together with significant heterogeneity, whereas stable SNPs were defined as variants showing concordant effects across both groups without significant heterogeneity. These SNP sets were then subjected to Multi-marker Analysis of GenoMic Annotation (MAGMA)-based gene-level analysis; therefore, “reversal genes” and “stable genes” refer to gene-level signals derived from the corresponding reversal and stable SNP sets, respectively, rather than genes selected directly by functional annotation. Variants consistently observed in both pre- and postmenopausal states were retained for downstream analysis. Gene-level mapping was performed using MAGMA [11], followed by functional annotation, pathway enrichment, and gene–concept network analysis [12,13]. These analyses were conducted independently in each dataset and subsequently integrated through cross-platform comparison to identify convergent biological pathways. After quality control and filtering, a total of 70 variants from the Illumina Infinium HumanExome BeadChip dataset (stable: 37; reversal: 33) and 946 variants from the Affymetrix Axiom Exome Array dataset (stable: 619; reversal: 327) were retained for MAGMA-based gene-level analysis.

### 2.2. Gene-Level Analysis Shows Different Signal Distributions of Reversal and Stable Signals

**Figure 2:**
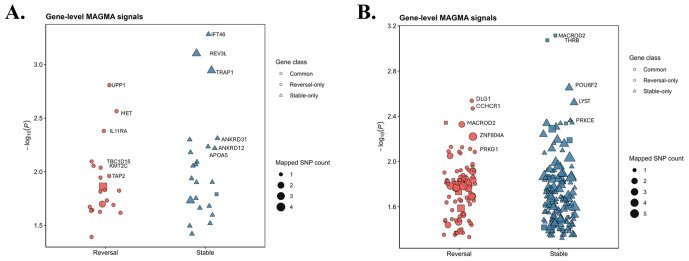
Gene-level MAGMA results for reversal and stable genes across Illumina Infinium HumanExome BeadChip and Affymetrix Axiom Exome Array datasets: (**A**) Illumina Infinium HumanExome BeadChip dataset. (**B**) Affymetrix Axiom Exome Array dataset. Gene-level association results are shown for reversal-only, stable-only, and common gene sets. Representative genes with relatively stronger signals included *IFT46*, *REV3L*, and *TRAP1* among stable genes, and *UPP1*, *MET*, and *IL11RA* among reversal genes. Some genes were shared between the datasets. Red indicates reversal-related genes, and blue indicates stable-related genes.

Gene-level association analysis revealed distinct distribution patterns between reversal and stable genes across both the Illumina Infinium HumanExome BeadChip and Affymetrix Axiom Exome Array datasets (Figure 2). Stable genes were more broadly distributed, with multiple genes showing moderate levels of association, whereas reversal genes tended to form more compact clusters. Several genes demonstrated relatively strong association signals. In the stable group, genes such as *IFT46*, *REV3L*, and *TRAP1* showed prominent signals, while in the reversal group, *UPP1*, *MET*, and *IL11RA* were among the most notable. A subset of genes was identified in both datasets.

### 2.3. Functional Annotation Highlights Category Shifts Between Stable and Reversal Genes

**Figure 3:**
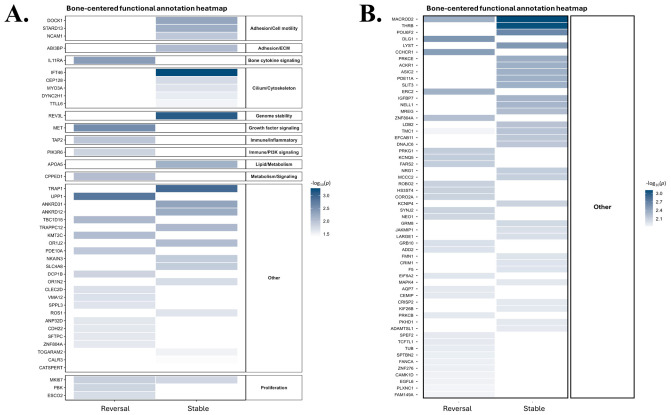
Functional annotation of reversal and stable genes across Illumina Infinium HumanExome BeadChip and Affymetrix Axiom Exome Array datasets: (**A**) Illumina Infinium HumanExome BeadChip dataset. (**B**) Affymetrix Axiom Exome Array dataset. Stable genes were more frequently assigned to cilium/cytoskeleton, intracellular transport, genome stability, and metabolism-related categories, whereas reversal genes were more frequently assigned to immune/inflammatory signaling and cytoskeleton-related categories. The Affymetrix Axiom Exome Array dataset contained a larger “Other” category. For readability, panel B displays the top 30 genes from each group ranked by −log10(*p*) to reduce label overlap.

To further characterize the identified genes, we performed functional annotation in both the Illumina Infinium HumanExome BeadChip and Affymetrix Axiom Exome Array datasets (Figure 3). Genes were classified into predefined functional categories, including adhesion/cell motility, extracellular matrix (ECM), cytokine signaling, cilium/cytoskeleton, genome stability, growth factor signaling, immune/inflammatory signaling, metabolism, and proliferation. Across both platforms, stable genes were predominantly represented in structural and signaling-related categories, including cilium/cytoskeleton organization, intracellular transport, genome stability, and lipid/metabolic regulation. In contrast, reversal genes showed relatively stronger representation in immune and inflammatory signaling as well as cytoskeletal remodeling-related processes. Overall, stable and reversal genes exhibited distinct functional category distributions across both datasets. Notably, the Affymetrix Axiom Exome Array dataset contained a substantially larger number of genes, resulting in a greater proportion being assigned to the “other” category.

### 2.4. Functional Enrichment Identifies Pathway-Level Differences Between Reversal and Stable Genes

**Figure 4:**
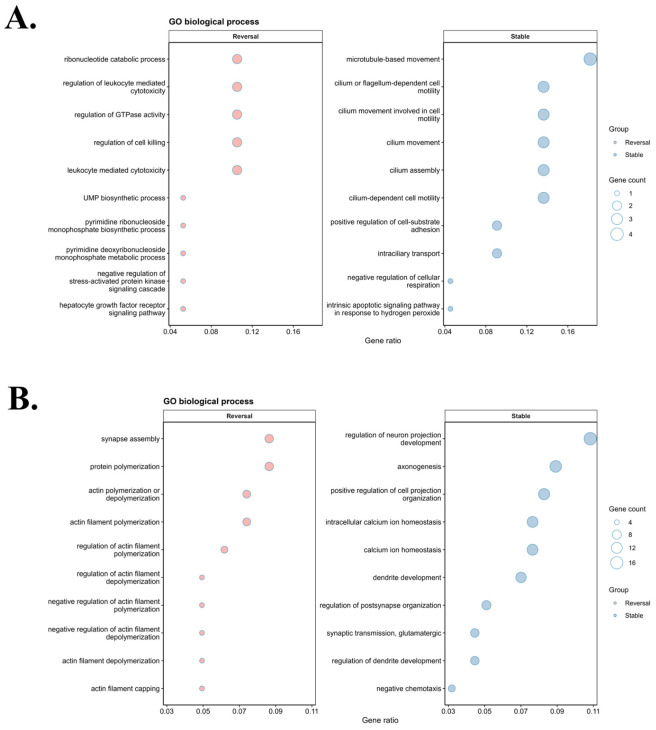
GO biological process enrichment of reversal and stable gene sets across Illumina Infinium HumanExome BeadChip and Affymetrix Axiom Exome Array datasets: (**A**) Illumina Infinium HumanExome BeadChip dataset. (**B**) Affymetrix Axiom Exome Array dataset. Stable genes were enriched for neuronal projection development, axonogenesis, dendrite development, intracellular calcium homeostasis, and cilium-related processes. Reversal genes were enriched for actin filament organization, protein polymerization, synapse assembly, leukocyte-mediated cytotoxicity, and regulation of cell killing. Comparable enrichment patterns were observed in both datasets.

Gene Ontology biological process (GO BP) enrichment analysis was performed for the reversal and stable gene sets in the Illumina Infinium HumanExome BeadChip and Affymetrix Axiom Exome Array datasets (Figure 4). In both datasets, stable genes were enriched for neuronal projection development, axonogenesis, dendrite development, intracellular calcium homeostasis, and cilium-related processes, including cilium assembly, cilium-dependent cell motility, and microtubule-based movement. Reversal genes were enriched for actin filament organization, protein polymerization, synapse assembly, leukocyte-mediated cytotoxicity, and regulation of cell killing. The overall enrichment patterns were similar across the two datasets. Complementary KEGG and Reactome enrichment analyses were also conducted to evaluate pathway-level consistency, and these results are provided in Figures S1 and S2.

### 2.5. Gene–Concept Networks Show Different Modular Architectures

**Figure 5:**
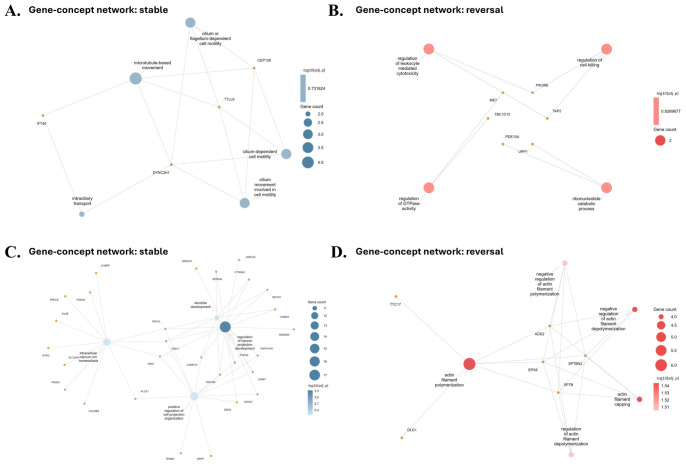
Gene–concept network analysis of reversal and stable gene sets across Illumina Infinium HumanExome BeadChip and Affymetrix Axiom Exome Array datasets: (**A**) Stable network (Illumina Infinium HumanExome BeadChip dataset). (**B**) Reversal network (Illumina Infinium HumanExome BeadChip dataset). (**C**) Stable network (Affymetrix Axiom Exome array dataset). (**D**) Reversal network (Affymetrix Axiom Exome array dataset). Gene–concept networks show the relationships between genes and enriched biological processes. Stable networks included terms related to neuronal development, intracellular transport, cilium-related processes, and calcium signaling, whereas reversal networks included actin cytoskeleton regulation, protein polymerization, and immune-related signaling. Actin filament organization and leukocyte-mediated cytotoxicity were present in the reversal networks. Orange nodes represent genes, whereas blue and red nodes represent enriched biological process terms in stable and reversal networks, respectively. Edges indicate gene–concept associations. Node size indicates gene count, and color intensity represents −log10(adjusted *p*-value).

To examine the relationships between genes and enriched biological processes, gene–concept networks were generated for each group and dataset (Figure 5). Stable gene networks contained multiple connections among terms related to neuronal development, intracellular transport, cilium-related processes, and calcium signaling. Reversal gene networks contained terms related to actin cytoskeleton regulation, protein polymerization, and immune-related signaling. Actin filament organization and leukocyte-mediated cytotoxicity were among the terms identified in the reversal networks. Differences in network structure were observed between stable and reversal gene sets across both datasets.

### 2.6. Cross-Platform Comparison of Shared Enriched Pathways

**Figure 6:**
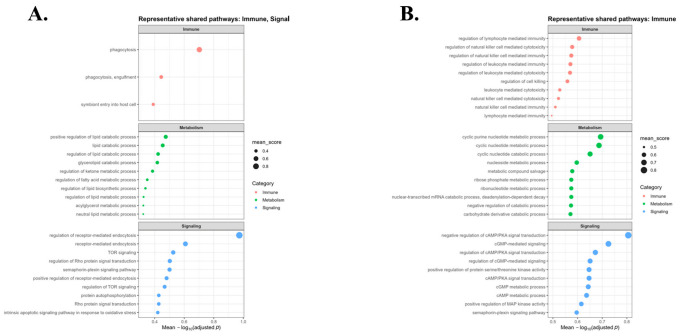
Cross-platform comparison of shared enriched pathways across Illumina Infinium HumanExome BeadChip and Affymetrix Axiom Exome Array datasets: (**A**) Stable shared pathways. (**B**) Reversal shared pathways. Shared enriched pathways were identified in both stable and reversal gene sets. Stable shared pathways included receptor-mediated signaling and lipid metabolic regulation. Reversal shared pathways included immune-related processes and cyclic nucleotide-related pathways, including cGMP-mediated signaling and regulation of cAMP/PKA signaling. Key shared reversal pathways are listed in Table 1.

Cross-platform comparison was performed to identify pathways shared between the Illumina Infinium HumanExome BeadChip and Affymetrix Axiom Exome Array datasets (Figure 6). Shared enriched pathways were observed in both stable and reversal gene sets. In the stable gene set, these included receptor-mediated signaling and lipid metabolic regulation. In the reversal gene set, shared pathways included immune-related processes, cyclic nucleotide metabolism, cGMP-mediated signaling, regulation of cAMP/PKA signaling, nitric oxide-dependent signaling, and kinase activity regulation. The shared pathways identified in the reversal gene set are listed in Table 1. Complementary cross-platform comparisons based on KEGG and Reactome pathway annotations are provided in Tables S1–S4.

## 3. Discussion

The central finding of this study is that low BMD-associated genetic signals across the menopausal transition become more interpretable when analyzed at the gene and pathway levels rather than at the level of individual SNP overlap. Importantly, this pattern was observed consistently across two independent genotyping platforms with distinct variant coverage, suggesting that the identified biological signals are unlikely to be platform-specific artifacts [8,10]. Reversal signals showed consistent convergence on cyclic nucleotide-related pathways, including cyclic nucleotide metabolism, cyclic adenosine monophosphate/cyclic guanosine monophosphate (cAMP/cGMP)–protein kinase A/protein kinase G (PKA/PKG) signaling, and the nitric oxide (NO)–guanylate cyclase–cGMP axis. In contrast, stable signals were more broadly distributed and were primarily associated with processes related to ionic homeostasis, cell–substrate adhesion, and structural maintenance. The enrichment of reversal signals in cyclic nucleotide-related pathways is biologically plausible, given the established roles of nitric oxide/cGMP and related signaling in osteoblast–osteoclast regulation, bone remodeling, mechanotransduction, and skeletal homeostasis [14]. By contrast, the broader distribution of stable signals may reflect more constitutive aspects of bone regulation that are less tightly coupled to the menopausal transition. Taken together, these results support a conceptual model in which low BMD across the menopausal transition may reflect a layered genetic architecture, comprising a stable maintenance axis and a menopause-associated transition axis. This framework extends beyond a simple binary classification of pre- and postmenopausal osteoporosis and instead suggests that female bone loss may be better understood as a continuous but dynamically reconfigured molecular spectrum. These patterns suggest a structured reorganization of regulatory pathways across the menopausal transition.

The limited overlap and lack of strong clustering at the individual SNP level are not unexpected, given differences in variant coverage across platforms and the polygenic architecture of BMD. Although we did not observe strong locus-level clustering, this pattern is consistent with the highly polygenic architecture typical of complex traits, where many weak-effect variants are expected to contribute jointly. From this perspective, gene- and pathway-level aggregation analyses can serve as a practical strategy to recover biologically coherent signals from dispersed variant-level associations [15,16,17]. Importantly, this reinforces the notion that biologically meaningful signals in complex traits may be better captured through aggregation across multiple variants rather than reliance on single-variant significance.

A notable observation is that reversal pathways converged on a phosphodiesterase (PDE)-centered cyclic nucleotide signaling axis, involving genes such as *PDE10A*, *PDE11A*, *PDE1A*, and *PRKG1*. This pattern suggests that the relative contribution of the NO–cGMP–PKG–PDE network may vary across the menopausal transition. Previous studies have implicated NO–cGMP signaling in osteoblast and osteocyte function, including mechanotransduction and bone remodeling, while PDEs modulate the amplitude and duration of these signals through cyclic nucleotide degradation. In this context, the observed reversal patterns are consistent with a potential shift in cyclic nucleotide signaling balance across menopause [14,18,19,20]. This interpretation is further supported by the role of estrogen. Estrogen is a key regulator of bone homeostasis and interacts functionally with NO–cGMP–PKG signaling pathways [21,22]. Under premenopausal conditions, cyclic nucleotide signaling may be relatively stable, whereas after menopause, estrogen decline may be associated with changes in the relative contribution of PDE–PRKG1-related signaling. Although the present study does not establish a direct regulatory relationship between estrogen and specific PDE isoforms, the repeated identification of PDE-centered pathways among reversal signals suggests that this axis may represent a candidate mechanism associated with menopausal transition in bone metabolism. Notably, these pathway-level patterns were consistently reproduced across two independent genotyping platforms with distinct variant coverage, supporting the robustness of biological convergence beyond SNP-level overlap.

Although the present pathway-level analysis does not provide cell-type-specific functional validation, recent osteoclast-focused functional genomic studies provide an important cellular framework for interpreting these findings. An osteoclast-specific expression quantitative trait locus (eQTL) study of BMD GWAS variants reported multiple regulatory associations in human osteoclasts, supporting the possibility that bone-density-associated variants may influence disease risk through osteoclast gene expression regulation [23]. In addition, RNA-seq analysis comparing human osteoclast-like cells with precursor peripheral blood mononuclear cells identified extensive transcriptomic changes during osteoclastogenesis and highlighted molecular pathways related to osteoclast differentiation and function [24]. More recent genome topology and transcriptomic analyses further suggest that bone-trait-associated loci may act through cell-type-specific chromatin loops and enhancer–promoter interactions in human osteoclasts [25]. These observations are relevant to the present study because reversal-associated pathways included actin filament organization, immune-related signaling, leukocyte-mediated cytotoxicity, and cyclic nucleotide signaling, all of which may intersect with osteoclast differentiation, cytoskeletal remodeling, migration, or resorptive activity. Although our analysis does not directly identify osteoclast-specific regulatory elements, the convergence of reversal signals on cytoskeletal and signaling pathways is consistent with the possibility that menopause-associated genetic reorganization may involve osteoclast-relevant regulatory programs.

In contrast, stable pathways appear to reflect a distinct and more constitutive biological layer. Rather than converging on a single dominant signaling axis, stable signals were distributed across processes such as ion transport, cell–substrate adhesion, and cytoskeletal organization. Collectively, these processes likely represent a baseline homeostatic framework related to mechanotransduction and skeletal maintenance across physiological states [26,27,28,29]. Accordingly, postmenopausal bone loss may involve accelerated regulatory changes that occur on top of a shared biological foundation characterized by ion homeostasis, extracellular matrix interaction, and structural stability.

Another important observation is that pathway-level reproducibility appeared more consistent than SNP-level overlap across the two array platforms, further supporting the robustness of biological convergence across datasets. This pattern suggests that distinct genetic variants may converge on shared genes or functional pathways, rather than requiring direct SNP-level replication [8,9]. In complex traits such as low BMD, gene- and pathway-level convergence may therefore offer a more informative level of biological interpretation than individual variant overlap [10]. Taken together, these findings support a layered interpretive framework in which stable signals may reflect a baseline susceptibility shared across menopausal status, whereas reversal signals may indicate transition-specific reorganization of regulatory pathways. From this perspective, low BMD can be viewed as a continuous molecular spectrum comprising both a shared maintenance axis and a menopause-associated transition axis [30,31].

Several limitations merit consideration. First, our pathway-level interpretations were based partly on overlaps derived from raw *p*-value-driven signals and should therefore be viewed as exploratory. Second, Gene Ontology categories are inherently redundant, and closely related biological processes may be captured by multiple overlapping terms [32]. Third, some MAGMA-derived gene-level signals were supported by only a small number of SNPs, which may reduce the robustness of these findings. Fourth, the lack of strong locus-level clustering limits direct locus-specific interpretation. Nevertheless, this limitation also motivated our emphasis on gene- and pathway-level convergence, which helped identify broader biologically relevant functional axes. Finally, the present study did not include experimental validation of the identified candidate pathways. Because this study was designed as a population-level genetic and pathway-based analysis, direct validation using quantitative polymerase chain reaction (qPCR), western blotting, or cell-based functional assays was beyond its scope. Therefore, the PDE–cyclic nucleotide signaling axis and other candidate pathways identified here should be interpreted as exploratory and hypothesis-generating rather than as definitive evidence of causal mechanisms. Future studies should validate the expression and functional relevance of these candidate genes and pathways in osteoclasts, osteoblasts, and osteocytes, particularly under estrogen-deficient or menopause-mimicking conditions. Integration with osteoclast-specific eQTL datasets, chromatin interaction maps, single-cell transcriptomics, and experimentally validated regulatory networks will help determine whether the reversal and stable pathways identified here act through osteoclasts, osteoblasts, osteocytes, or other bone-related cellular compartments.

In conclusion, low BMD in women is associated with a broadly shared clinical outcome across menopausal status, but it does not appear to be governed by a single molecular program. Some signals are preserved as stable biological processes across both states, whereas others appear to be reorganized after menopause toward cyclic nucleotide-related signaling pathways. These findings support the view of female osteoporosis as a continuous molecular spectrum rather than as two entirely distinct conditions. Although exploratory, this study provides a cross-platform validated framework for interpreting genetic signals across physiological transitions. More broadly, our findings suggest that apparent discrepancies at the SNP or gene level may reflect shared pathway-level biology at the network level, supporting a multi-level interpretation beyond variant-level concordance.

## 4. Materials and Methods

### 4.1. Study Population and Operational Definition of Low BMD

Menopausal status and the operational definition of low BMD were applied as previously described [3,4]. Briefly, postmenopausal women were defined as participants with confirmed menopause in the Korean Genome and Epidemiology Study (KoGES) cohort, whereas premenopausal women comprised non-menopausal participants from the same cohort. Individuals with factors that could affect bone mineral density, including steroid use, fracture history, relevant medication use, or excessive alcohol consumption, were excluded. Low BMD was defined using a quantitative ultrasound (QUS)-based operational approach based on T-scores and Z-scores derived from speed-of-sound measurements at the distal radius and midshaft tibia (T-score < −2.5 and Z-score < −2.0). These criteria were used for research purposes and were not intended to correspond directly to the WHO/ISCD clinical diagnostic criteria for osteoporosis based on central dual-energy X-ray absorptiometry (DXA) [33]. The study was approved by the Institutional Review Board of Dankook University (Institutional Review Board No. 2018-08-004; 10 September 2018).

### 4.2. GWAS Summary Statistics Processing and Quality Control

Genome-wide association summary statistics (GWAS) were obtained separately for the premenopausal and postmenopausal groups. For each dataset, variants were required to include the SNP identifier, chromosome, base-pair position, effect allele, non-effect allele, effect size (β), standard error (SE), minor allele frequency, sample size, and association *p*-value. To ensure robust and comparable signals across datasets, SNP-level quality control was applied prior to downstream analyses. Variants were filtered using the following criteria: minor allele frequency (MAF) ≥ 0.01 and Hardy–Weinberg equilibrium *p* ≥ 1 × 10^−6^. To remove unstable association estimates, SNPs with SE > 1 or an absolute effect size |β| > 2 were excluded. In addition, variants showing no evidence of association in either dataset (*p* ≥ 0.05 in both groups) were excluded from subsequent comparative analyses.

### 4.3. Allele Harmonization and Identification of Shared Variants

To ensure consistent interpretation of allelic effects across datasets, allele harmonization was performed using the premenopausal dataset as the reference. For SNPs with discordant allele coding between the premenopausal and postmenopausal datasets, effect estimates in the postmenopausal dataset were aligned by inverting the sign of the effect size (β × −1), while retaining the corresponding SE and *p*-value. Strand-ambiguous SNPs (A/T or C/G) were excluded when allele orientation could not be reliably determined. After harmonization, only SNPs present in both datasets were retained. This intersection defined the set of directly comparable variants used for subsequent heterogeneity and directionality analyses.

### 4.4. Heterogeneity Testing and Direction-Based SNP Classification

To quantify differences in genetic effects between premenopausal and postmenopausal states, a heterogeneity test was performed for each shared SNP using the following Z-statistic:

$$Z=\frac{\beta_{\mathrm{pre}}-\beta_{\mathrm{post}}}{\sqrt{SE_{\mathrm{pre}}^{2}+SE_{\mathrm{post}}^{2}}}$$

Two-sided heterogeneity *p*-values were calculated from the standard normal distribution:

$$P_{\mathrm{het}}=2\times (1-\Phi (|Z|))$$

To retain biologically relevant variants, SNPs were required to show nominal evidence of association in at least one group (*p* < 0.05 in either premenopausal or postmenopausal analysis).

Shared SNPs were then classified according to directionality and heterogeneity patterns. Reversal SNPs were defined as variants showing significant heterogeneity (P_het_ < 0.05) together with opposite effect directions between groups (β_“pre” × β_“post” < 0), with at least one effect size exceeding a minimal magnitude (|β| ≥ 0.1). Stable SNPs were defined as variants associated with low BMD in both groups (*p* < 0.05 in both) without significant heterogeneity (*P*_het_ ≥ 0.05). Variants with negligible effect sizes in both groups or lacking evidence of association were excluded. These thresholds were used for exploratory classification and were not intended to represent genome-wide significance.

### 4.5. Preparation of MAGMA Input Files

For downstream gene-level analysis, reversal and stable SNP sets were exported separately in a MAGMA-compatible format, including SNP identifier, chromosome, base-pair position, association *p*-value, and sample size [11]. For each retained SNP, the *p*-value used as input to MAGMA was defined as the minimum of the two GWAS *p*-values obtained from the premenopausal and postmenopausal analyses. The minimum of the two GWAS *p*-values was used while preserving prior classification into reversal and stable groups. In addition, an SNP location file containing the SNP identifier, chromosome, and base-pair position was generated for gene annotation.

### 4.6. Gene-Level and Pathway Analysis

Gene-level association analysis was performed using MAGMA v1.10 [11]. SNPs were mapped to genes based on genomic position using the National Center for Biotechnology Information (NCBI) human genome build 37.3 gene location annotation file aligned to the Genome Reference Consortium Human Build 37/human genome version 19 (GRCh37/hg19) reference build. LD structure was modeled using the 1000 Genomes Project Phase 3 East Asian reference panel. Separate gene-based analyses were conducted for the reversal and stable SNP sets using the corresponding SNP-wise association *p*-values. The sample size assigned to each SNP was matched to the GWAS result from which the corresponding *p*-value was derived. MAGMA output included gene identifiers, the number of mapped SNPs, Z-statistics, and gene-level *p*-values.

Genes showing nominal evidence of association in the MAGMA analysis, defined as gene-level *p* < 0.05 before multiple-testing correction, were retained separately for the reversal and stable groups for downstream functional analysis. Reversal and stable gene sets were not defined using GO terms or pathway annotations. Instead, SNPs were first classified into reversal or stable groups based on cross-menopausal effect direction and heterogeneity, and these SNP sets were then independently subjected to MAGMA-based gene-level analysis. GO, KEGG, and Reactome analyses were subsequently performed only after MAGMA-based gene selection and were used to interpret the biological characteristics of the resulting gene sets. Gene identifiers were converted from Entrez Gene IDs to official gene symbols using the org.Hs.eg.db annotation database in R. Shared and group-specific genes were identified by direct comparison of Entrez Gene IDs.

Functional enrichment analysis was performed in R (version 4.5.3) using Bioconductor version 3.22 packages. GO BP enrichment was conducted using clusterProfiler version 4.18.4 with org.Hs.eg.db version 3.22.0 as the annotation reference, Kyoto Encyclopedia of Genes and Genomes (KEGG) pathway analysis using enrichKEGG, and Reactome pathway analysis using ReactomePA version 1.54.0. Reversal and stable gene sets were analyzed independently. Multiple testing correction was performed using the Benjamini–Hochberg method. Because relatively few genes entered enrichment analysis, the results were interpreted as exploratory.

### 4.7. Visualization and Analytical Framework

Enrichment results were visualized in R using ggplot2 version 4.0.2. For each database, top-ranked pathways were summarized using horizontal bar plots ordered by −log_10_(*p*-value). Gene-level MAGMA results were visualized using jitter plots of −log_10_(P), with symbols indicating reversal-specific, stable-specific, and shared genes.

A category-based heatmap was generated by grouping selected genes into biologically relevant functional classes, including cilium/cytoskeleton, adhesion/cell motility, immune/inflammatory signaling, growth factor signaling, lipid/metabolism, proliferation, and genome stability. Gene–concept network plots were constructed from the top GO BP terms using the igraph version 2.2.2 and ggraph version 2.2.2 packages.

Downstream analyses were designed to compare reversal signals, defined as variants with directionally discordant effects between premenopausal and postmenopausal analyses, and stable signals, defined as variants with concordant effects across menopausal strata. Gene-level aggregation and pathway enrichment were performed separately for these groups to distinguish menopause-sensitive and menopause-independent biological programs.

## Figures and Tables

**Table 1:** Shared enriched pathways in the reversal gene set across Illumina Infinium HumanExome BeadChip and Affymetrix Axiom Exome Array datasets. Representative shared pathways and associated genes identified in the Illumina Infinium HumanExome BeadChip and Affymetrix Axiom Exome Array datasets.

Pathway	exo *p*-Value	affy *p*-Value	exo Genes	affy Genes
negative regulation of cAMP/PKA signal transduction	0.0278	0.0064	*PDE10A*	*PDE11A*, *PDE1A*
cGMP-mediated signaling	0.0376	0.0116	*PDE10A*	*PDE11A*, *PRKG1*
cyclic purine nucleotide metabolic process	0.0425	0.0147	*PDE10A*	*CACNB4*, *PDE1A*
cyclic nucleotide metabolic process	0.0434	0.0153	*PDE10A*	*CACNB4*, *PDE1A*
regulation of cAMP/PKA signal transduction	0.0463	0.0174	*PDE10A*	*PDE11A*, *PDE1A*

affy: Affymetrix Axiom Exome Array, exo: Illumina Infinium HumanExome BeadChip.

## Data Availability

The raw genotype data used in this study were obtained from the Korean Genome and Epidemiology Study (KOGES) under a data-use license and cannot be shared publicly due to institutional and ethical restrictions. Summary-level statistical results generated during this study are available from the corresponding author upon reasonable request.

## Supplementary Materials

The following supporting information can be downloaded at: https://www.mdpi.com/article/10.3390/ijms27104447/s1.

## Abbreviations

The following abbreviations are used in this manuscript:

BMD	bone mineral density
LD	linkage disequilibrium
GO BP	Gene Ontology biological process
NO	nitric oxide
PDE	phosphodiesterase
QUS	quantitative ultrasound
KoGES	Korean Genome and Epidemiology Study
KEGG	Kyoto Encyclopedia of Genes and Genomes
SNP	single-nucleotide polymorphism
MAGMA	Multi-marker Analysis of GenoMic Annotation
cAMP	cyclic adenosine monophosphate
cGMP	cyclic guanosine monophosphate
PKA	protein kinase A
PKG	protein kinase G
eQTL	expression quantitative trait locus
qPCR	quantitative polymerase chain reaction
DXA	dual-energy X-ray absorptiometry
NCBI	National Center for Biotechnology Information
GRCh	Genome Reference Consortium Human
hg19	human genome version 19
MAF	minor allele frequency
