# Supplementary material for: Pathway-Level Reorganization of Genetic Signals Associated with Low Bone Mineral Density Across the Menopausal Transition
Source: Int J Mol Sci. 2026 May 15;27(10):4447. doi: 10.3390/ijms27104447 (PMC13207153; doi:10.3390/ijms27104447)
Supplement: Supplementary file 1 [file ijms-27-04447-s001.zip › Supplementary Figures S1 & S2.pdf]

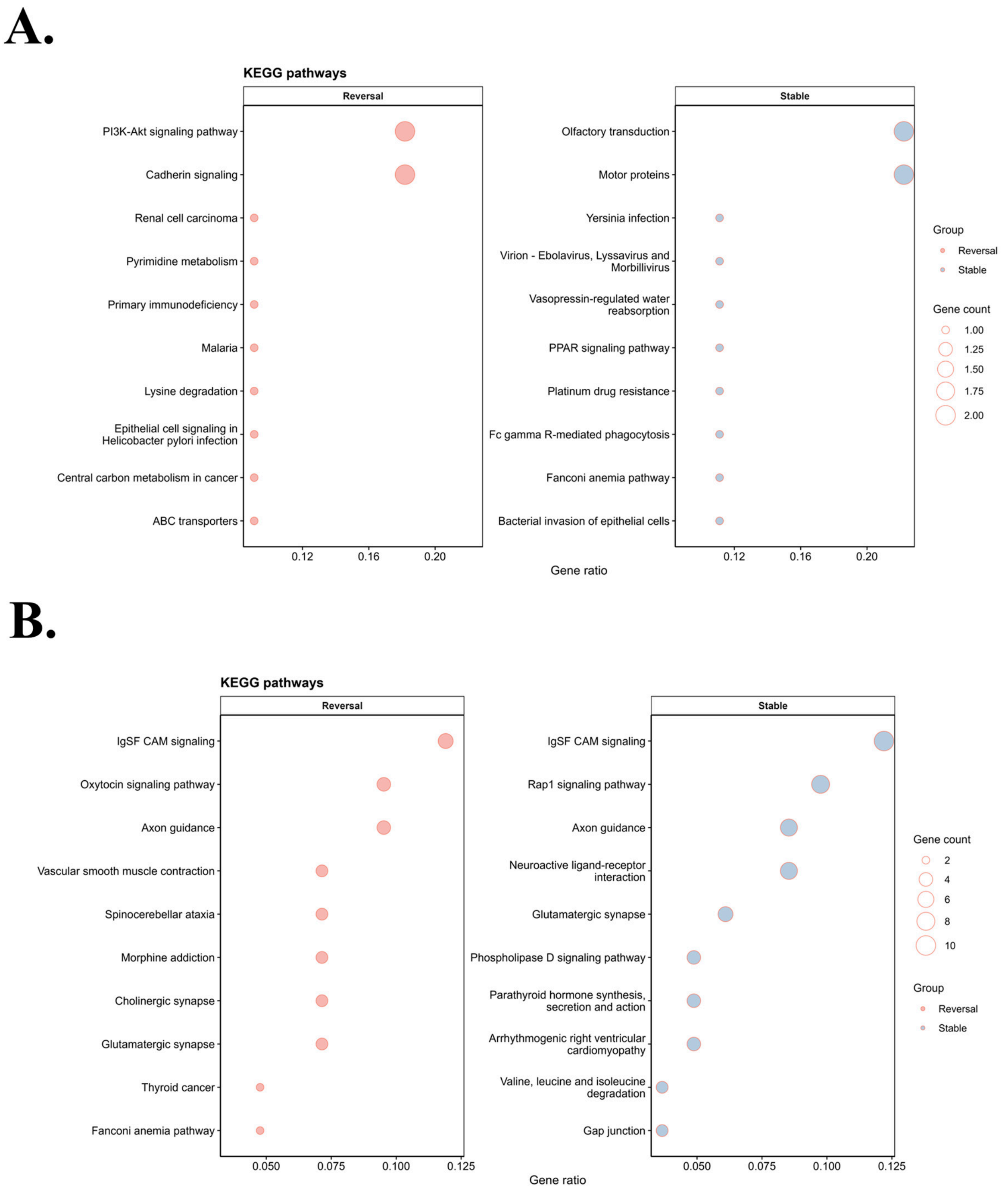

Figure S1. KEGG biological process enrichment of reversal and stable gene sets across Illumina Infinium HumanExome BeadChip and Affymetrix Axiom Exome Array datasets. (**A**) Illumina Infinium HumanExome BeadChip dataset. (**B**) Affymetrix Axiom Exome Array dataset.

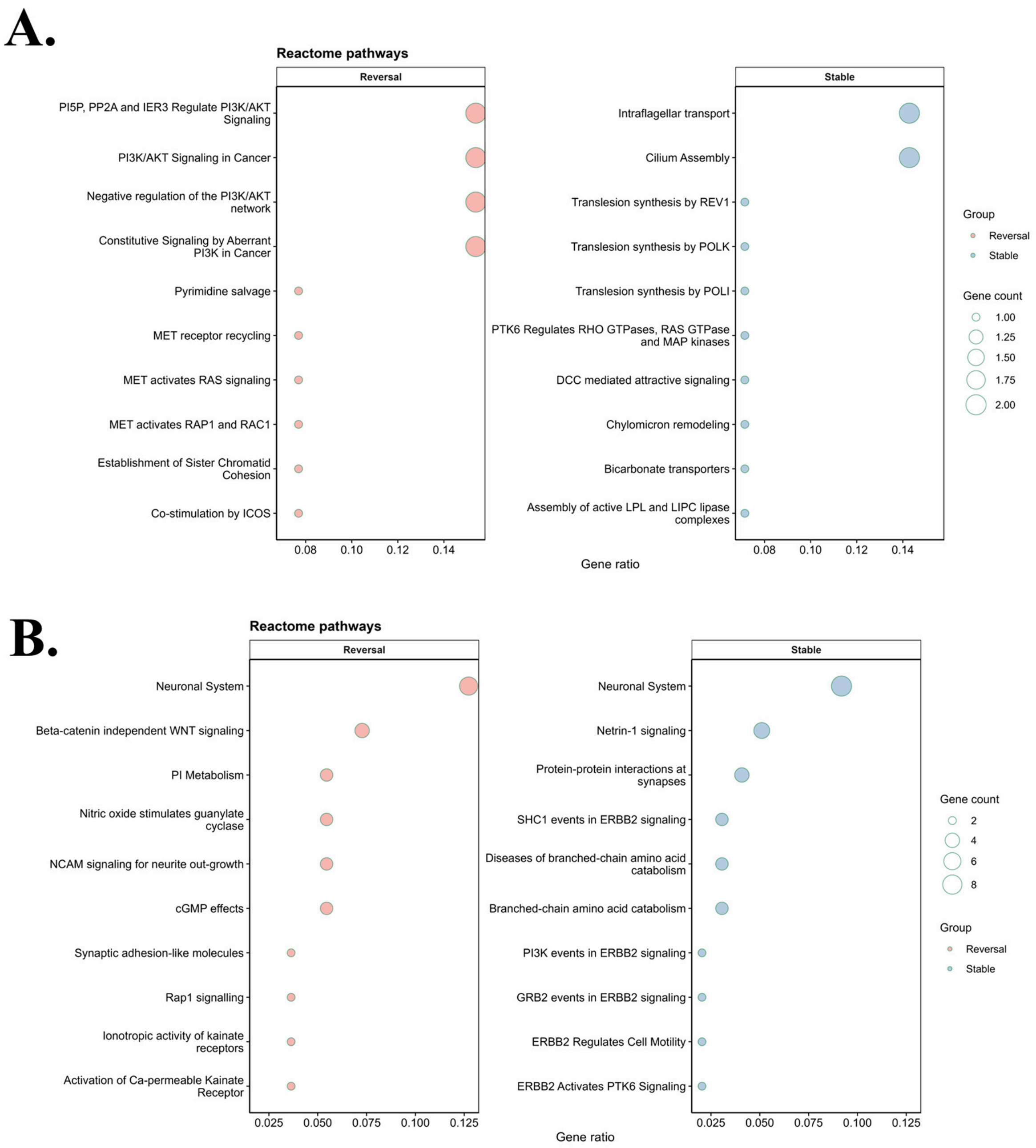

Figure S2. Reactome biological process enrichment of reversal and stable gene sets across Illumina Infinium HumanExome BeadChip and Affymetrix Axiom Exome Array datasets. (**A**) Illumina Infinium HumanExome BeadChip dataset. (**B**) Affymetrix Axiom Exome Array dataset.
