# Supplementary material for: Pathway-Level Reorganization of Genetic Signals Associated with Low Bone Mineral Density Across the Menopausal Transition
Source: Int J Mol Sci. 2026 May 15;27(10):4447. doi: 10.3390/ijms27104447 (PMC13207153; doi:10.3390/ijms27104447)
Supplement: Supplementary file 1 [file ijms-27-04447-s001.zip › Supplementary Tables S1-S4.pdf]

Table S1. Shared KEGG pathways in reversal.

No.	Pathway	exo *p*-Value	affy *p*-Value	exo Gene ID(s)	affy Gene ID(s)
1	Morphine addiction	0.1018	0.0077	10846	50940/5136/5579
2	Cholinergic synapse	0.1270	0.0146	146850	56479/5579/146850
3	Platelet activation	0.1384	0.1090	146850	5592/146850
4	Purine metabolism	0.1404	0.1119	10846	50940/5136
5	Apelin signaling pathway	0.1526	0.1297	146850	22808/146850
6	Phospholipase D signaling pathway	0.1617	0.0287	146850	11069/22808/146850
7	Oxytocin signaling pathway	0.1666	0.0048	146850	785/57118/5579/146850
8	Adrenergic signaling in cardiomyocytes	0.1666	0.0313	146850	785/11069/146850
9	cGMP-PKG signaling pathway	0.1785	0.1700	146850	5592/146850
10	Hepatocellular carcinoma	0.1824	0.1764	4233	83439/5579

Note: No shared KEGG reversal pathway met dual raw $p < 0.05$ or dual raw $p < 0.1$.

Table S2. Shared KEGG pathways in stable.

No.	Pathway	exo *p*-Value	affy *p*-Value	exo Gene ID(s)	affy Gene ID(s)
1	Cell adhesion molecule (CAM) interaction	0.1436	0.1644	4684	3680/5789/57689
2	Regulation of actin cytoskeleton	0.2020	0.0522	1793	3680/10788/1956/1902/3071
3	Shigellosis	0.2183	0.1803	1793	5581/3710/1956/51196
4	IgSF CAM signaling	0.2531	0.000262	4684	9223/8633/6586/9863/137970/5789/6709/57689/23426/4008
5	Bacterial invasion of epithelial cells	0.0725	0.4974	1793	1496
6	Motor proteins	0.0139	0.5089	53904/79659	55083/3834
7	Focal adhesion	0.1789	0.5334	1793	3680/1956
8	Fc gamma R-mediated phagocytosis	0.0903	0.5791	1793	5581
9	Prion disease	0.2351	0.6985	4684	3710/2903
10	Yersinia infection	0.1250	0.7051	1793	64283
11	Phagosome	0.1394	0.7466	79659	81035
12	Salmonella infection	0.2145	0.8901	79659	3071
13	Parkinson disease	0.2298	0.9082	10131	3710
14	Olfactory transduction	0.0659	0.9093	26740/138882	83988/123041
15	Pathways of neurodegeneration - multiple diseases	0.3770	0.9279	10131	3710/2903

Note: No shared KEGG stable pathway met dual raw $p < 0.05$ or dual raw $p < 0.1$.

Table S3. Shared Reactome pathways in reversal.

No.	Pathway	exo *p*-Value	affy *p*-Value	exo Gene(s)	affy Gene(s)
1	cGMP effects	0.0184	6.00e-05	*PDE10A*	*PDE11A/PDE1A/PRKG1*
2	Nitric oxide stimulates guanylate cyclase	0.0252	1.60e-04	*PDE10A*	*PDE11A/PDE1A/PRKG1*
3	Co-stimulation by ICOS	0.0127	0.0526	*PIK3R6*	*PIK3R6*
4	Synthesis of PIPs at the plasma membrane	0.0597	0.0277	*PIK3R6*	*SYNJ2/PIK3R6*
5	Semaphorin interactions	0.0706	0.0380	*MET*	*PLXNC1/MYH11*
6	Regulation of MITF-M-dependent genes involved in cell cycle and proliferation	0.0206	0.0846	*MET*	*TCF7L1*
7	PI Metabolism	0.0930	0.0080	*PIK3R6*	*SYNJ2/MTMR10/PIK3R6*
8	Platelet homeostasis	0.0973	0.0091	*PDE10A*	*PDE11A/PDE1A/PRKG1*

Note: Two shared Reactome reversal pathways met dual raw $p < 0.05$: **cGMP effects** and **Nitric oxide stimulates guanylate cyclase**.

Table S4. Shared Reactome pathways in stable.

No.	Pathway	exo *p*-Value	affy *p*-Value	exo Gene(s)	affy Gene(s)
1	Signal transduction by L1	0.0259	0.0142	*NCAM1*	*ITGA9/EGFR*
2	Netrin-1 signaling	0.0511	3.00e-05	*DOCK1*	*UNC5C/SLIT3/UNC5D/DSCAM/TRPC5*
3	Signaling by PTK6	0.0653	0.0805	*DOCK1*	*EGFR/NRG1*
4	Signaling by Non-Receptor Tyrosine Kinases	0.0653	0.0805	*DOCK1*	*EGFR/NRG1*
5	Sensory processing of sound by outer hair cells of the cochlea	0.0653	0.0805	*MYO3A*	*TMC1/SPTAN1*
6	RHOC GTPase cycle	0.0839	0.0232	*STARD13*	*FMNL2/ARHGEF28/DLC1*
7	Bicarbonate transporters	0.0124	0.0840	*SLC4A8*	*SLC4A4*

Note: One shared Reactome stable pathway met dual raw $p < 0.05$: **Signal transduction by L1**.
